# The unmet demand for point-of-care ultrasound among general pediatricians: a cross-sectional survey

**DOI:** 10.1186/s12909-021-03072-1

**Published:** 2022-01-03

**Authors:** Anelah McGinness, Margaret Lin-Martore, Newton Addo, Ashkon Shaahinfar

**Affiliations:** 1 Department of Graduate Medical Education, PGY-3 resident, University of California San Francisco Benioff Children’s Hospital Oakland, 747 52nd Street, Suite 245, Oakland, CA 94609 USA; 2 Departments of Emergency Medicine and Pediatrics, University of California San Francisco, 5150 16th St, Box 0632, San Francisco, CA 94143 USA; 3 Departments of Medicine and Emergency Medicine, 1001 Potrero Ave, Box 1220, San Francisco, CA 94143 USA; 4 Departments of Emergency Medicine and Pediatrics, University of California San Francisco Benioff Children’s Hospital Oakland, University of California San Francisco, 747 52nd Street, ED Trailer 3, Oakland, CA 94609 USA

**Keywords:** Ultrasound, Pediatrics, POCUS

## Abstract

**Background:**

Point-of-care ultrasound (POCUS) is a noninvasive bedside tool with many pediatric applications but is not currently a formal part of pediatric training and practice. Formal surveys of general pediatricians regarding POCUS training are lacking. We aimed to quantify the baseline ultrasound experience and training needs of general pediatricians and pediatric residents across different practice settings.

**Methods:**

In 2020, we sent an online survey to 485 current faculty, residents, and graduates from an urban pediatric academic medical center in Northern California. Pediatric subspecialists were excluded. Survey questions about baseline experience, comfort, and perceived usefulness of 20 common POCUS applications were developed by two POCUS experts using existing literature. Chi-squared analysis was used to compare residents versus attendings and to compare attendings practicing in inpatient versus outpatient versus mixed settings.

**Results:**

Response rate was 20% (98/485). Compared to attendings (*n* = 73), residents (*n* = 25) endorsed more exposure to POCUS in medical school (32% vs 5%, *p* = 0.003) and residency (12% vs 5%, *p* = 0.003). Respondents endorsed low comfort with POCUS (mean 1.3 out of 5 on Likert scale). Of 20 procedural and diagnostic applications, respondents identified abscess drainage, bladder catheterization, soft tissue, neck, advanced abdominal, and constipation as most useful. Overall, 50% of pediatricians (and 70% of pediatric residents) responded that there were opportunities to use POCUS multiple times a week or more in their clinical practice.

**Conclusions:**

There is an unmet demand for POCUS training among general pediatricians and trainees in our study. Although the majority of respondents were not POCUS users, our results could guide future efforts to study the role of POCUS in general pediatrics and develop pediatric curricula.

**Supplementary Information:**

The online version contains supplementary material available at 10.1186/s12909-021-03072-1.

## Background

Point-of-care ultrasound (POCUS) is a portable, increasingly affordable, non-invasive tool with many pediatric applications supported by previous research [1]. In the pediatric emergency medicine literature, POCUS has been shown to improve diagnostic accuracy [2], aid in procedural guidance [3], and decrease length of stay [4]. The diagnostic information obtained by POCUS can be used to reduce the need for other imaging modalities and other more invasive tests, aiding in radiological stewardship and high value care [5, 6]. Compared to adults, small children offer the additional advantage of often being easier to scan, as their size facilitates easy penetration by ultrasound waves with high-frequency transducers, producing superior resolution images [7]. However, in spite of these advantages for pediatric use, many of which would likely be of benefit to the general pediatric practitioner, POCUS is not currently a standard part of pediatric training or practice.

POCUS is no longer a tool only for pioneers and early adopters. As of 2014, 62% of medical schools reported integrating POCUS into their curricula [8]. At the pediatric fellowship level, POCUS has become a standard part of pediatric emergency medicine training [9, 10]. POCUS is also becoming increasingly utilized in pediatric critical care [11, 12] and neonatology [13, 14] fellowships. Compared to pediatric subspecialty and adult counterparts, POCUS remains underutilized in general pediatric training. In a 2019 survey by Reaume et al., 37.5% of internal medicine programs and 43.5% of combined medicine-pediatric programs reported formal POCUS curricula while only 12.4% pediatric programs reported offering a formal ultrasound curriculum [15]. Furthermore, the authors note that this may represent an overestimate due to potential response-bias, as those with ultrasound programs may have been more likely to respond to a survey about ultrasound curricula.

Various authors have published recent review articles listing POCUS scans that would be most applicable to the practice of a general pediatrician in the acute care setting based on extrapolation from internal medicine, emergency medicine, and pediatric emergency medicine [1, 12, 16–18]. However, little is known regarding general pediatricians’ baseline POCUS training, their perceptions of POCUS, and which specific applications would be most useful in daily clinical practice from the perspective of a general pediatrician. Focused needs assessments guiding development of roadmaps for POCUS curricula based on responses from internal medicine and family medicine residents and faculty have been conducted [19–28]. Corresponding surveys among pediatricians to guide the development of pediatric POCUS curricula are lacking.

We hypothesized that general pediatricians would identify a variety of specific areas of practice where they could apply POCUS, that trainees would have more exposure to POCUS training than attendings, and that the ultrasound applications perceived to be useful by pediatricians would differ from their adult or emergency medicine counterparts. We performed a cross-sectional survey asking pediatric residents and general pediatric attendings across the inpatient and outpatient settings about their exposure to ultrasound training, their baseline comfort with specific ultrasound applications, and their perceived usefulness of specific ultrasound scans in their clinical practice.

## Methods

### Study population

In September 2020, we sent an online survey to a total of 485 pediatric residents, pediatric urgent care faculty, pediatric hospitalist faculty, primary care pediatric faculty, and recent graduates (graduating classes of 2010 - 2019) from an urban, community-based, pediatric academic medical center in Northern California. Current pediatric faculty and residents were identified using employee email lists maintained by the supervisors of these respective groups. A list of the names and email addresses of the resident alumni maintained by chief residents was used to identify all who had graduated from pediatric residency from 2010 to 2019. Fellows and attendings practicing in pediatric subspecialties were excluded. We included those who had completed a hospitalist or adolescent medicine fellowship.

### Survey and data collection

An electronic survey was distributed via email to all 485 identified participants. Additionally, a link to the survey was posted on social media – specifically to a 383 member private Facebook (Facebook, Menlo Park, CA) group consisting of graduates from the pediatric residency and to private Slack (Slack, San Francisco, CA) and GroupMe (Microsoft, New York, NY) channels. Many current faculty members are also members of these social media groups. Fliers with a quick response (QR) code that linked to the survey were also distributed during resident teaching conferences and posted in both faculty and resident workrooms to which key or badge access was required. The survey was created in Qualtrics (Qualtrics, Provo, UT) based on review of prior needs assessments designed for family medicine and internal medicine [21, 22, 26, 28]. Specific pediatric POCUS applications were selected based on published reviews describing the scans most pertinent to pediatric POCUS [1, 12, 16] and based on input from 2 of the authors (AS, MLM), who are experts in pediatric POCUS. We requested feedback from a small advisory group of medical education experts from a variety of specialties at our institution to improve survey clarity and breadth. We asked participants to score their baseline comfort (from 1 = extremely uncomfortable to 5 = extremely comfortable) and the perceived usefulness (from 1 = I would never use this to 5 = extremely useful) of 8 procedural and 12 diagnostic pediatric POCUS applications on 5-point Likert-type scale. The survey instrument is available in supplemental material, (Additional file 1).

### Data analysis

Descriptive statistics were tabulated in Microsoft Excel (Version 16.50) from survey results to determine characteristics of respondents and their mean responses to survey questions. Differences in mean self-reported usefulness or comfort scores were assessed with ANOVA and Chi-squared tests or Fisher’s exact test were used to compare categorical response variables, as appropriate. All statistical analysis were performed using R version 3.6 (R Core Team, 2020). In subgroup analyses, we compared attendings (*n* = 73) vs residents (*n* = 25), and we compared inpatient attendings (*n* = 17) vs outpatient attendings (*n* = 32) vs attendings who practice in both the inpatient and outpatient setting (“mixed”, *n* = 24). We included urgent care attendings who practice in the hospital setting in the “mixed” category.

This study was deemed exempt by the University of California, San Francisco Institutional Review Board. Each participant provided informed consent prior to starting the survey and all responses were voluntary; participants were not required to answer all questions.

## Results

### Respondent practice setting and POCUS utilization

A total of 25 residents and 73 attendings completed our survey, with a total response rate of 20% (98 of 485). Of the attendings and recent graduates who responded, the majority practiced in an urban setting (73%), did not have access to a POCUS machine (82%), and did not use POCUS for clinical decisions (94%). The anticipated future practice settings for the 25 residents who responded (Table 1) were similar to the attendings, with the majority (88%) planning to practice in outpatient pediatrics. A higher proportion of residents (52%) than attendings (6%) reported using POCUS for clinical decisions during their residency (*p* < 0.0001, Table 1). Of the four pediatric attendings in our sample who endorsed using POCUS for clinical decisions, all of them practiced either full-time or part-time in the inpatient setting. Two of these four endorsed no formal training. Of the two who used POCUS but endorsed no formal training, one worked in rural setting, and one worked in both an academic center and a community hospital. The two who endorsed receiving formal training did so longitudinally in fellowship (hospital medicine) and in an ultrasound bootcamp/conference, respectively.

**Table 1:** Participant Characteristics

Level of Training	% (n)
Pediatric Resident	26% (25)
Attending/Faculty	74% (73)
Total	100% (98)
Attending/Faculty characteristics (*n* = 73)*	% (n)
Pediatric hospitalist medicine (inpatient general pediatrics)	40% (29)
Pediatric primary care (outpatient general pediatrics)	56% (41)
Adolescent medicine	5% (4)
Urgent care	26% (19)
Solo or two physician practice	5% (4)
Academic medical center	42% (31)
Community hospital	23% (17)
Federally Qualified Health Center	26% (19)
Group practice or HMO	41% (30)
Rural	4% (3)
Suburban	26% (19)
Urban	70% (51)
Access to POCUS machine - No	82% (58)
Bill for POCUS - Yes	0% (0)
Use POCUS for clinical decisions - Yes	6% (4)
POCUS training in medical school – Yes	5% (4)
POCUS training in residency - Yes	5% (4)
Residents (*n* = 25)*	% (n)
Pediatric hospitalist medicine (inpatient general pediatrics)	44% (11)
Pediatric primary care (outpatient general pediatrics)	56% (14)
Procedural pediatric sub-specialty	8% (2)
Adolescent medicine	4% (1)
Urgent care	36% (9)
Non-procedural pediatric sub-specialty	4% (1)
Use POCUS for clinical decisions - Yes	52% (13)
POCUS training in medical school – Yes	32% (8)
POCUS training in residency - Yes	12% (3)

* Respondents were able to select more than one response for all sections

### Baseline exposure to POCUS training and comfort

Compared to attendings, residents endorsed more exposure to POCUS in medical school (32% vs 5%, *p* = 0.003) and in residency (12% vs 5%, *p* = 0.003, Table 1). On average, pediatricians in our sample endorsed feeling “extremely” to “somewhat uncomfortable” with POCUS – corresponding to a mean 1.2 out of 5 for diagnostic POCUS and 1.4 out of 5 for procedural POCUS on a 5-point Likert scale. More residents than attendings endorsed being at least “4 - somewhat comfortable” with diagnostic POCUS (17% vs 0%, *p* = 0.004). There were no significant differences in comfort with procedural POCUS between residents and attendings. Among attendings, only 1 inpatient attending endorsed feeling at least “4 - somewhat comfortable” with procedural POCUS, but there were no statistically significant differences between inpatient vs outpatient vs mixed attendings in baseline comfort with POCUS.

### Perceived usefulness of POCUS

**Fig. 1:**
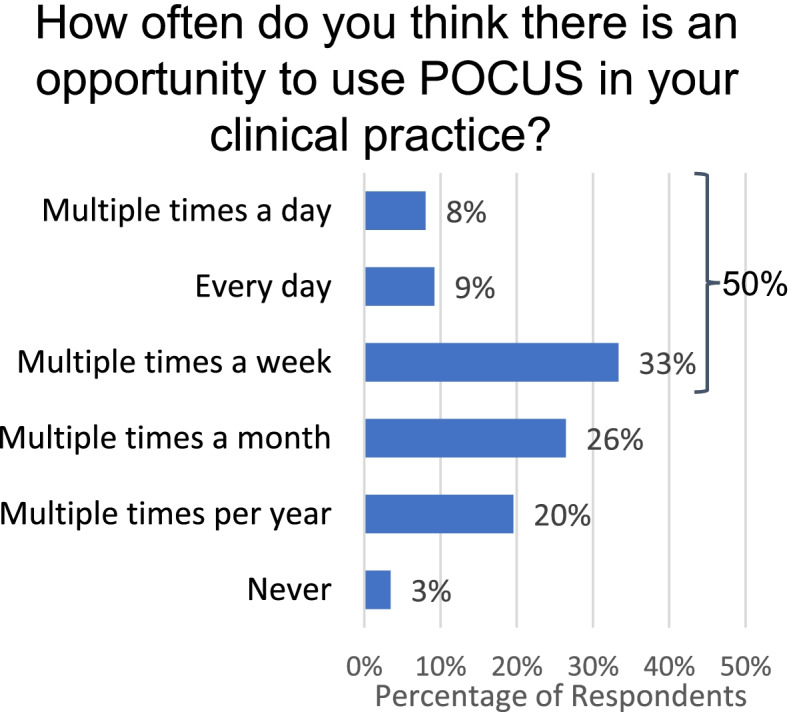
Perceived opportunities for use of POCUS by general pediatricians. Providers responded to the question “How often do you think there is an opportunity to use POCUS in your clinical practice?” (Percentages were calculated as number of responses divided by total *n* = 98)

When asked “how often do you think there is an opportunity to use POCUS in your practice?” 50% of all respondents responded that there were opportunities to use POCUS “multiple times a week” or more in their clinical practice (Fig. 1). Compared to attendings, a higher proportion of residents responded that they would use POCUS “multiple times a week” or more (70% vs 45%, *p* = 0.08), although this was not statistically significant. Among attendings, a higher percentage of inpatient attendings responded that they would use POCUS “multiple times a week” or more (60%), followed by mixed attendings (45%), and last by outpatient attendings (37%), although when groups were compared, differences were not statistically significant (*p* = 0.33). See (Additional file 2) to view subgroup analysis.

**Fig. 2:**
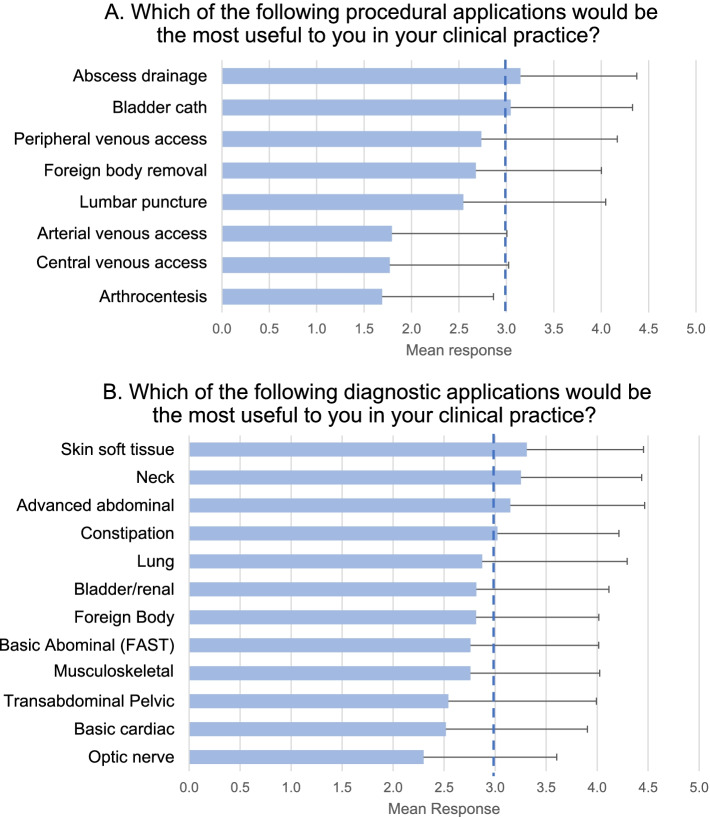
**a** Procedural POCUS applications and their perceived utility to general pediatricians. Pediatricians were asked the question “Which of the following procedural applications would be the most useful to you in your clinical practice?” They rated the following procedural applications on a 5 point Likert scale, (where 1 = I would never use this 2 = somewhat useful 3 = useful 4 = very useful 5 = extremely useful): abscess drainage, bladder volume measurement (i.e. prior to cath), peripheral vascular access, foreign body removal, lumbar puncture (including post-LP hematoma), arterial vascular access, Central vascular access, arthrocentesis. Note that the majority of respondents to this question were not formally trained in POCUS. **b** Diagnostic POCUS applications and their perceived utility to general pediatricians. Pediatricians were asked the question “Which of the following diagnostic applications would be the most useful to you in your clinical practice?” They rated the following diagnostic POCUS applications on a 5-point Likert scale (where 1 = I would never use this 2 = somewhat useful 3 = useful 4 = very useful 5 = extremely useful): skin and soft tissue (cellulitis or abscess), neck (lymphadenopathy vs abscess vs mass), advanced abdominal (appendicitis, intussusception, hypertrophic pyloric stenosis, cholecystitis), constipation (transrectal diameter to assess rectal stool burden), lung (pneumothorax, pneumonia, bronchiolitis, pleural effusion/empyema), genitourinary (bladder volume, hydronephrosis), foreign bodies (soft tissue), basic abdominal (abdominal free fluid), musculoskeletal (long bone and clavicle fractures, skull fractures, joint effusion), transabdominal pelvic (early pregnancy detection, IUD placement confirmation), focused cardiac exam (pericardial effusions, global cardiac function), optic nerve measurement (papilledema). Note that the majority of respondents to this question were not formally trained in POCUS

Of the 8 procedural applications, abscess drainage and bladder volume measurement prior to catheterization were scored at least “3 - useful” on average by all respondents. Of the 12 diagnostic applications, “skin and soft tissue (cellulitis or abscess)”, “neck (lymphadenopathy vs abscess vs mass)”, “advanced abdominal (appendicitis, intussusception, hypertrophic pyloric stenosis, cholecystitis)”, and “constipation (transrectal diameter to assess rectal stool burden)” were scored as “3 - useful” on average by all respondents. Average scoring of all surveyed applications, from most to least useful, is shown in Fig. 2. Subgroup analysis comparing how applications were ranked by various groups is available in (Additional file 2).

Compared to attendings, residents overall assigned significantly higher average usefulness scores across procedural POCUS applications (mean 3.53 vs 2.10, *p* < 0.001) and diagnostic POCUS applications (mean 3.77 vs 2.57, *p* < 0.001). Compared to outpatient attendings, those practicing in the inpatient setting or in the mixed setting assigned significantly higher usefulness scores for procedural POCUS (mean usefulness score out of 5 on a Likert scale, 2.71 inpatient vs 2.23 mixed vs 1.69 outpatient, *p* < 0.001) and for diagnostic POCUS (mean score out of 5 on a Likert scale, 2.88 inpatient vs 2.72 mixed vs 2.30 outpatient, *p* < 0.001). See (Additional file 2) to view subgroup analysis.

### Perceived barriers to POCUS

**Fig. 3:**
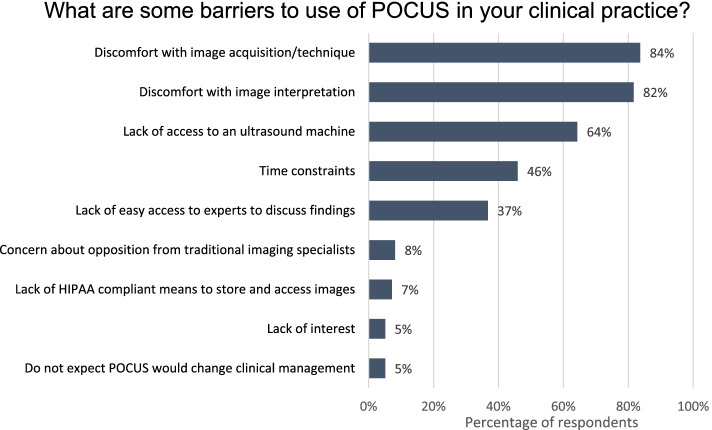
Perceived barriers to POCUS use by pediatricians. Pediatricians were asked the question “What are some barriers to use of POCUS in your clinical practice?” Respondents were allowed to select more than one response. (Percentages calculated as number of responses divided by total *n* = 98)

A majority of pediatricians in our sample noted the following barriers to use of POCUS in clinical practice: discomfort with image acquisition/technique, discomfort with image interpretation, lack of access to an ultrasound machine (Fig. 3). Pediatricians also cited time constraints (46%) and lack of easy access to experts to discuss findings (37%) as barriers.

## Discussion

The majority of general pediatricians and pediatric trainees in our study responded that there were frequent opportunities to use POCUS in their clinical practice. However, most did not have the training, comfort, and tools necessary to perform POCUS.

This study represents one of few surveys of POCUS utility for general pediatricians both in training and in practice [15, 29]. Residents universally scored POCUS more highly in terms of usefulness and, in fact, in subgroup analysis 25% of pediatric residents responded that they could identify opportunities to use POCUS multiple times a day in their clinical practice. Importantly, the majority of pediatric trainees are using POCUS in their clinical practice (52%), though few pediatric attendings are (6%), thus raising questions of supervision, safety and quality assurance. This disparity in POCUS training between trainees and faculty is found similarly in needs assessments of adult specialties [19, 22, 24, 30] and is likely reflective of national trends as medical schools increasingly integrate POCUS into their curricula [8]. This also underscores the urgency that pediatric residencies follow the trend led by medical schools and adult specialties in developing ultrasound curricula [25, 30, 31]. Given the technical skill required for POCUS [32], new practitioners need skilled preceptors to oversee and hone this important clinical skill. We did not ask trainees in our study if they were using POCUS unsupervised, but currently the trainees at our institution only have access to POCUS machines in the emergency department, neonatal intensive care unit, and pediatric intensive care unit – all departments where they have access to trained POCUS preceptors. Currently, trainees in our institution are unable to practice POCUS in outpatient clinics or on the general pediatrics floors where they do not have access to POCUS machines. Although the residents in our study showed more interest in POCUS on average than practicing attendings, it is just as important that continuing medical education and accreditation be developed and made available beyond residency given that the vast majority of pediatric providers did not receive formal training in POCUS and may need to supervise trainees who are already using POCUS in clinical care.

Outpatient attendings perceived POCUS as less useful, on average, than those practicing in the inpatient setting. However, as seen in prior studies, perceived utility may change with increased exposure [27]. Supporting this notion, family medicine is also heavily outpatient and clinical practice overlaps with pediatrics, yet other studies have found that graduates from family medicine programs where POCUS is taught continue to use POCUS in their clinical practice [33]. Prior review articles on pediatric POCUS have focused on the role of POCUS in the practice of the pediatric hospitalist [1, 12, 16–18, 34]. However, the majority of pediatric residents in our study were interested in POCUS, and over half of them planned to practice outpatient general pediatrics. As more pediatric residencies include ultrasound in their training, the role of POCUS in the pediatric outpatient setting may grow substantially.

The pediatricians in our study ranked the usefulness of specific POCUS applications differently than in prior studies focusing on internal medicine and family medicine. For example, in prior studies of internal medicine physicians, central line placement, paracentesis, and thoracentesis were the most highly ranked procedural POCUS applications [20, 35]. However, in our study, central line placement was ranked among the least useful, along with arterial venous access and arthrocentesis. For diagnostic POCUS, cardiac POCUS, lung (particularly pleural effusion), DVT, and abdominal free fluid tend to be ranked most useful in prior surveys of adult practitioners [20, 21, 35–37], while for the pediatricians in our study, abdominal free fluid and cardiac applications were again ranked least useful. Interestingly, pediatricians ranked neck (lymph node vs abscess), advanced abdominal (appendicitis, intussusception, hypertrophic pyloric stenosis, cholecystitis) among the most useful diagnostic applications, studies which tend to be considered more advanced by POCUS experts [12, 16]. While the POCUS literature for pediatric appendicitis [2, 38] and intussusception [39, 40] is growing, there is currently limited evidence to support the use of POCUS for differentiation of neck masses [41–43]. Constipation was also ranked as a useful diagnostic application by pediatricians; however, there are currently only a handful of studies on the validity of measuring transrectal diameter in the diagnosis of pediatric constipation [44–46]. It should be noted that the majority of respondents in our study were not POCUS users, and thus it is unlikely that technical difficulty of each scan or the diagnostic advantages or limitations were taken into account in their ranking. However, although these advanced applications may not have as broad support in the literature, their perceived potential clinical utility in our study provides important insight into research gaps and ways in which POCUS could potentially impact the clinical practice and diagnostic capabilities among general pediatricians in the future. Designs of ultrasound curricula for pediatricians should combine the clinical needs identified by pediatricians with guidance from POCUS educational experts who are well versed in the evidence to support pediatric POCUS.

The barriers to POCUS use for the pediatricians in our study were similar to those described in studies of family medicine, internal medicine and pediatric emergency medicine [25, 47, 48], including discomfort with performing and interpreting POCUS scans, time constraints, lack of machines (with some preferring hand held machines), and lack of easy access to guidance or quality assurance from POCUS experts. Other studies in pediatric emergency medicine [23, 48, 49] have additionally noted to varying degrees institutional barriers to advancement of POCUS such as implementation of billing for POCUS, systems for archiving POCUS scans, consultant acceptance of non-radiology performed ultrasound, and funding for POCUS training. Credentialing and assessment of competency are also barriers which pediatric emergency medicine has recently started to address [32] and which emergency medicine physicians are still standardizing [50]. As more general pediatricians become trained in POCUS applications, additional research is required to address these important issues in POCUS.

### Limitations

Our study had a number of limitations. Our sample was limited to residents and pediatricians currently working at or trained by one center in one state, limiting generalizability. Our low response rate at 20%, although comparable to other published surveys [23, 26, 51], may be a source of bias as it is possible that physicians and trainees with an interest or background in ultrasound may have been more likely to respond to a study about ultrasound, thus overestimating the demand for POCUS training and skewing our results. Indeed, in our barriers question, very few respondents (5%) endorsed a lack of interest in POCUS. There are few prior needs assessments specific to pediatrics, thus although our survey was based on published literature, it was not validated and it was not test-piloted in our study population prior to full dissemination. In our survey, we asked respondents to rate individual applications based on potential clinical utility on a Likert scale by asking what would be “the most useful to you in your clinical practice?”. It is possible that “usefulness” can mean different things to different people. To some it may mean frequency of use, to others it may mean sensitivity/specificity, and to others it may mean the degree to which it changes clinical management. The majority of the physicians in our study did not have formal POCUS training, therefore we were not able to ask more specifically about most used applications or about applications most likely to change management. Because most respondents lacked first-hand knowledge of the required skill level and training, test characteristics, limitations, and clinical utility typical of each scan, their rankings of usefulness in our study, although providing important insights from the perspective of the general pediatric practitioner, are not alone sufficient to design a roadmap for pediatric POCUS curricula on a national level. It is possible, as seen in other studies, that as pediatricians get more exposure to POCUS they may rank POCUS applications differently [27, 33]. Similar to prior studies [27] those with more experience with POCUS (residents) also showed more interest in using POCUS and gaining further training in POCUS.

### Future directions

The American Medical Association’s position statement regarding privileging states that, ‘ultrasound imaging is within the scope of practice of appropriately trained physicians” and that physicians should qualify for privileging if they possess appropriate training as specified by their respective specialty association [52]. Although medical societies representing pediatric emergency medicine [10], internal medicine [53, 54], and family medicine [31] have published consensus statements providing guidance for POCUS curricula for their respective specialties, no such guidelines have yet been published by pediatric medical organizations. Although our data suggested a demand for POCUS training, there are many unanswered questions as how this training should be conducted, how to introduce basic versus advanced applications, and how new pediatric POCUS users should be supervised and assessed for competency. Future models of training and supervision might include those led by pediatric subspecialists such as pediatric emergency medicine, radiology, cardiology, or critical care as well as uptraining of general pediatric POCUS champions (e.g. through POCUS fellowship). Teletechnology approaches that include both real-time teleguidance and asynchronous quality assurance may augment the reach of general pediatric POCUS champions who have completed a fellowship or certification process [55, 56].

We hope that our cross-sectional survey can serve as a starting point for future needs assessments, either on a larger scale, or for use by other pediatric hospital groups who are trying to develop POCUS curricula for their pediatric practitioners. Future needs assessments focusing on general pediatric attendings who are already using POCUS in their clinical practice, such as a Delphi approach, may provide valuable perspective on which applications are most useful, although given how few there are (6% of our sample) such a study may need to be national in scope. Further assessment of pediatricians and pediatric resident ultrasound experience and training needs on a national level are warranted to determine the extent to which POCUS should be a standard part of pediatric residency curricula and which applications should be prioritized.

## Conclusions

There is an unmet demand for point-of-care ultrasound training for the pediatricians in our study. Pediatric residents had more experience using POCUS and perceived POCUS as more useful than attendings. General pediatricians who worked full or part time in the inpatient setting, including urgent care, were more significantly likely to view POCUS as useful than outpatient attendings. Given the tools and the training, general pediatricians would be better able to identify POCUS applications that would have an impact on their clinical practice and the opportunities to use them.

## Supplementary Information

**Additional file 1.** Pediatric point-of-care ultrasound survey instrument.

**Additional file 2.** Subgroup analysis, comparison of responses of attendings vs residents, comparison of responses of inpatient vs mixed vs outpatient attendings.

## Data Availability

The datasets used and/or analyzed during the current study are available from the corresponding author on reasonable request.

## Abbreviations

POCUS: Point-of-care ultrasound
DVT: Deep Venous Thrombosis
FAST: Focused assessment with sonography in trauma
QR code: Quick response code
