# Supplementary material for: The unmet demand for point-of-care ultrasound among general pediatricians: a cross-sectional survey
Source: BMC Med Educ. 2022 Jan 3;22:7. doi: 10.1186/s12909-021-03072-1 (PMC8722332; doi:10.1186/s12909-021-03072-1)
Supplement: Supplementary file 1 — Additional file 1. Pediatric point-of-care ultrasound survey instrument. [file 12909_2021_3072_MOESM1_ESM.pdf]

Point-of-Care Ultrasound Needs Assessment for Pediatricians Survey

Start of Block: Informed Consent

Q1
Welcome to the Point-of-Care Ultrasound (POCUS) for pediatricians needs assessment!

We are conducting a needs assessment to determine the training needs for Point-of-Care/bedside ultrasound (POCUS) general pediatricians. We are interested in surveying general pediatricians working in the inpatient and outpatient setting at UCSF Benioff Children's Hospital Oakland (BCHO) and graduates from the BCHO pediatric residency who are working as general pediatricians in the inpatient and outpatient setting.

We are interested in understanding to what degree general pediatricians have had POCUS training, use POCUS in their daily practice, desire training in various POCUS applications, and barriers/support for implementation of POCUS training. For this study, you will be asked questions about prior POCUS training and the role POCUS might have in your clinical practice. The risks of participating in this study are minimal. Your responses will be anonymous and confidential.

If you choose to be in the study, you will complete an online survey. It will take about **10 minutes** to complete.

Your participation in this research is voluntary. You can skip questions that you do not want to answer or stop the survey at any time. The survey is anonymous, and no one will be able to link your answers back to you. Please do not include your name or other information that could be used to identify you in the survey responses.

The Principal Investigator of this study can be contacted at ashkon.shaahinfar@ucsf.edu with questions about this study. If you have questions or concerns about your rights as a research participant, you can call the UCSF Institutional Review Board at 415-476-1814.

By clicking the button below, you acknowledge:
Your participation in the study is voluntary. You are above 18 years of age. You are aware that you may choose to terminate your participation at any time for any reason.
Thank you,
Anelah McGinness, MD. UCSF Benioff Children's Hospital Oakland GME, anelah.mcginness@ucsf.edu
Ashkon Shaahinfar, MD. UCSF Benioff Children's Hospital Oakland Pediatric Emergency, ashkon.shaahinfar@ucsf.edu

- ○ I consent, begin the study (1)
- ○ I do not consent, I do not wish to participate (2)

End of Block: Informed Consent

Start of Block: Population characteristics

Q2 What level of training are you currently in?

- ○ Pediatric resident (1)
- ○ Fellow (4)
- ○ Attending/faculty (5)

Display This Question:

If What level of training are you currently in? = Fellow

Or What level of training are you currently in? = Attending/faculty

Q3 At this time, we are interested in the training needs of general pediatricians. Which of the following best describes your current clinical practice? Select all that apply.

- ☐ Pediatric hospitalist medicine (inpatient general pediatrics) (1)
- ☐ Urgent care (11)
- ☐ Pediatric primary care (outpatient general pediatrics) (2)
- ☐ Adolescent medicine (8)
- ☐ Procedural pediatric sub-specialty: (4) ____
- ☐ Non-procedural pediatric sub-specialty: (12) ____

Q4 Which of the following best describes your current practice type?

- ☐ Solo or two physician practice (1)
- ☐ Group practice or HMO (7)
- ☐ Academic medical center (3)
- ☐ Federally Qualified Health Center (6)
- ☐ Community hospital (4)

Q5 Which of the following best describes your current practice setting?

- ○ Urban (1)
- ○ Suburban (5)
- ○ Rural (2)

Display This Question:

If What level of training are you currently in? != Fellow

And What level of training are you currently in? != Attending/faculty

Q6 In what area(s) of pediatrics do you plan to practice upon graduation? Select all that apply.

- ☐ Pediatric hospitalist medicine (inpatient general pediatrics) (1)
- ☐ Pediatric primary care (outpatient general pediatrics) (2)
- ☐ Urgent care (11)
- ☐ Adolescent medicine (8)
- ☐ Procedural pediatric sub-specialty: (4) ____
- ☐ Non-procedural pediatric sub-specialty: (12) ____

End of Block: Population characteristics

Start of Block: Current training/exposure/comfort

Q7 Do you currently have access to a Point-of-Care Ultrasound (POCUS) machine for use in your clinical practice?

- ○ Yes (1)
- ○ No (2)

Q8 Do you regularly bill for performing POCUS scans?

- ○ Yes (1)
- ○ No (2)

Q9 Do you use POCUS in your current clinical practice to inform clinical decisions?

- Yes (1)
- No (2)

Q10 If you have had formal training in POCUS, in what setting did you receive your education? (select all that apply)

- No POCUS training (14)
- Medical school - preclinical elective (1)
- Medical school - clinical elective (3)
- Residency - dedicated ultrasound elective (4)
- Residency - longitudinal scanning sessions (5)
- Fellowship - dedicated ultrasound elective (6)
- Fellowship - longitudinal scanning sessions (7)
- Ultrasound fellowship (13)
- Ultrasound conference/boot camp (10)
- Other training (describe below) (15)

Display This Question:

If If you have had formal training in POCUS, in what setting did you receive your education? (select... = Other training (describe below)

Q11 If you have had training in settings other than those listed above, please describe who trained you, the setting, and the stage of training you were in:

____

____

____

____

____

X→

Q12 Select the approximate quantity of POCUS scans you have performed on a real patient with (or without) the supervision of an experienced POCUS mentor:

	0 patient scans (0)	1-10 patient scans (1)	10-50 patient scans (2)	50+ patient scans (3)
Supervised (1)	○	○	○	○
Unsupervised (2)	○	○	○	○

End of Block: Current training/exposure/comfort

Start of Block: Current comfort and desired training

Q13 The following questions ask about your **current level of comfort** with procedural and diagnostic POCUS applications.

Page Break

Q14 Indicate your current level of comfort with the following procedural POCUS applications:

	Extremely uncomfortable (1)	Somewhat uncomfortable (2)	Neither comfortable nor uncomfortable (3)	Somewhat comfortable (4)	Extremely comfortable (5)
Central vascular access (1)	○	○	○	○	○
Peripheral vascular access (2)	○	○	○	○	○
Arterial vascular access (3)	○	○	○	○	○
Lumbar puncture (including post-LP hematoma) (5)	○	○	○	○	○
Arthrocentesis (6)	○	○	○	○	○
Bladder volume measurement (ie prior to cath) (7)	○	○	○	○	○
Abscess drainage (10)	○	○	○	○	○
Foreign body removal (11)	○	○	○	○	○
Other (12)	○	○	○	○	○

Q15 Indicate your current level of comfort with the following diagnostic POCUS applications:

	Extremely uncomfortable (1)	Somewhat uncomfortable (2)	Neither comfortable nor uncomfortable (3)	Somewhat comfortable (4)	Extremely comfortable (5)
Lung (Pneumothorax, pneumonia, bronchiolitis, pleural effusion/empyema) (1)	○	○	○	○	○
Focused cardiac exam (pericardial effusions, global cardiac function) (3)	○	○	○	○	○
Foreign bodies (soft tissue) (4)	○	○	○	○	○
Skin and soft tissue (cellulitis or abscess) (5)	○	○	○	○	○
Neck (lymphadenopathy vs abscess vs mass) (6)	○	○	○	○	○
Optic nerve measurement (papilledema) (7)	○	○	○	○	○
Constipation (transrectal diameter to assess rectal stool burden) (8)	○	○	○	○	○
Musculoskeletal (long bone and clavicle fractures, skull fractures, joint effusion) (9)	○	○	○	○	○
Genitourinary (bladder volume, hydronephrosis) (10)	○	○	○	○	○

Basic abdominal (abdominal free fluid) (13)	○	○	○	○	○
Advanced abdominal (appendicitis, intussusception, hypertrophic pyloric stenosis, cholecystitis) (21)	○	○	○	○	○
Transabdominal pelvic (early pregnancy detection, IUD placement confirmation) (22)	○	○	○	○	○
Other (23)	○	○	○	○	○

Q16 How would you rate your current overall comfort in using POCUS for **procedural** applications?

○ Extremely uncomfortable (18)

○ Somewhat uncomfortable (19)

○ Neither comfortable nor uncomfortable (20)

○ Somewhat comfortable (21)

○ Extremely comfortable (22)

Q17 How would you rate your current overall comfort in using POCUS for **diagnostic** applications?

- ○ Extremely uncomfortable (20)
- ○ Somewhat uncomfortable (21)
- ○ Neither comfortable nor uncomfortable (22)
- ○ Somewhat comfortable (23)
- ○ Extremely comfortable (24)

Page Break

Q18 The following questions ask about the **clinical utility** of various procedural and diagnostic POCUS applications pediatricians.

Page Break

Q19 Which of the following procedural applications would be the most useful to you in your clinical practice?

	I would never use this (1)	Somewhat useful (2)	Useful (3)	Very useful (4)	Extremely useful (5)
Central vascular access (1)	o	o	o	o	o
Peripheral vascular access (2)	o	o	o	o	o
Arterial vascular access (3)	o	o	o	o	o
Lumbar puncture (including post-LP hematoma) (5)	o	o	o	o	o
Arthrocentesis (6)	o	o	o	o	o
Bladder volume measurement (ie prior to cath) (7)	o	o	o	o	o
Abscess drainage (10)	o	o	o	o	o
Foreign body removal (11)	o	o	o	o	o
Other (12)	o	o	o	o	o

Q20 Which of the following diagnostic applications would be the most useful to you in your clinical practice?

	I would never use this (1)	Somewhat useful (2)	Useful (3)	Very useful (4)	Extremely useful (5)
Lung (Pneumothorax, pneumonia, bronchiolitis, pleural effusion/empyema) (1)	○	○	○	○	○
Focused cardiac exam (pericardial effusions, global cardiac function) (3)	○	○	○	○	○
Foreign bodies (soft tissue) (4)	○	○	○	○	○
Skin and soft tissue (cellulitis or abscess) (5)	○	○	○	○	○
Neck (lymphadenopathy vs abscess vs mass) (6)	○	○	○	○	○
Optic nerve measurement (papilledema) (7)	○	○	○	○	○
Constipation (transrectal diameter to assess rectal stool burden) (8)	○	○	○	○	○
Musculoskeletal (long bone and clavicle fractures, skull fractures, joint effusion) (9)	○	○	○	○	○
Genitourinary (bladder volume, hydronephrosis) (10)	○	○	○	○	○
Basic abdominal (abdominal free fluid) (13)	○	○	○	○	○

Advanced abdominal (appendicitis, intussusception, hypertrophic pyloric stenosis, cholecystitis) (21)	○	○	○	○	○
Transabdominal pelvic (early pregnancy detection, IUD placement confirmation) (22)	○	○	○	○	○
Other (23)	○	○	○	○	○

Q21 How often do you think there is an opportunity to use POCUS in your clinical practice?

○ Multiple times a day (1)

○ Every day (2)

○ Multiple times a week (3)

○ Multiple times a month (16)

○ Multiple times per year (17)

○ Never (18)

End of Block: Current comfort and desired training

Start of Block: Attitudes and barriers

Q22 What are some barriers to use of POCUS in your clinical practice?

- ▢ Lack of access to an ultrasound machine (1)
- ▢ Lack of HIPAA compliant means to store and access images (4)
- ▢ Discomfort with image acquisition/technique (5)
- ▢ Discomfort with image interpretation (6)
- ▢ Lack of easy access to experts to discuss findings (8)
- ▢ Concern about opposition from traditional imaging specialists (11)
- ▢ Do not expect POCUS would change clinical management (12)
- ▢ Time constraints (3)
- ▢ Lack of interest (10)
- ▢ Other (15)

Display This Question:

If What are some barriers to use of POCUS in your clinical practice? = Other

Q23 If you selected "other", please describe the barriers to POCUS in your clinical practice

____

____

____

____

____

End of Block: Attitudes and barriers

Start of Block: Incentive
