# Supplementary material for: The unmet demand for point-of-care ultrasound among general pediatricians: a cross-sectional survey
Source: BMC Med Educ. 2022 Jan 3;22:7. doi: 10.1186/s12909-021-03072-1 (PMC8722332; doi:10.1186/s12909-021-03072-1)
Supplement: Supplementary file 2 — Additional file 2. Subgroup analysis, comparison of responses of attendings vs residents, comparison of responses of inpatient vs mixed vs outpatient attendings. [file 12909_2021_3072_MOESM2_ESM.pptx]

## Slide 1
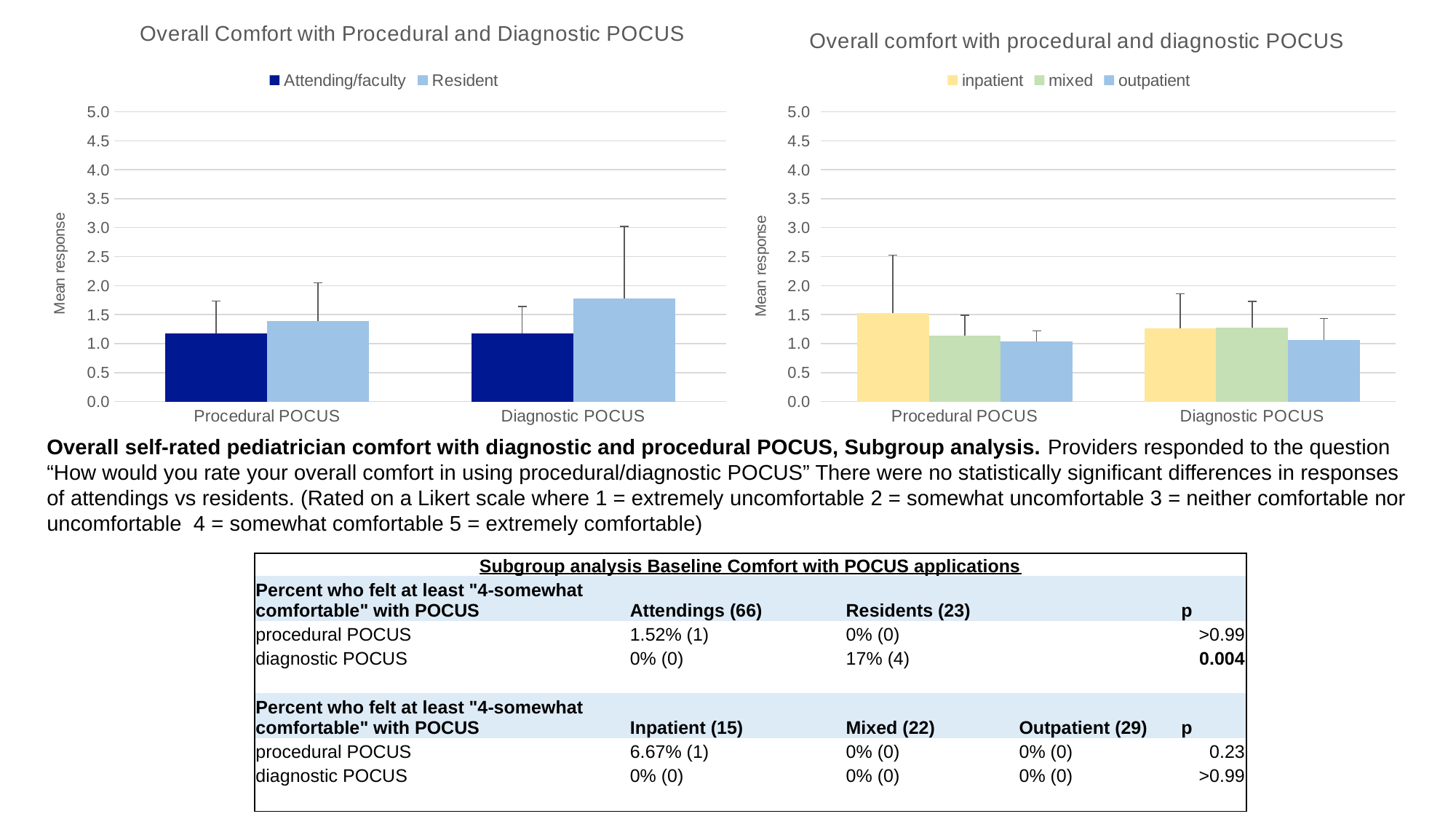

### Chart: Overall Comfort with Procedural and Diagnostic POCUS
| Category | | |
|---|---|---|
| Procedural POCUS | 1.1818181818181819 | 1.391304347826087 |
| Diagnostic POCUS | 1.1791044776119404 | 1.7826086956521738 |
### Chart: Overall comfort with procedural and diagnostic POCUS
| Category | | | |
|---|---|---|---|
| Procedural POCUS | 1.5333333333333334 | 1.1363636363636365 | 1.0344827586206897 |
| Diagnostic POCUS | 1.2666666666666666 | 1.2727272727272727 | 1.0666666666666667 |Overall self-rated pediatrician comfort with diagnostic and procedural POCUS, Subgroup analysis. Providers responded to the question “How would you rate your overall comfort in using procedural/diagnostic POCUS” There were no statistically significant differences in responses of attendings vs residents. (Rated on a Likert scale where 1 = extremely uncomfortable 2 = somewhat uncomfortable 3 = neither comfortable nor uncomfortable 4 = somewhat comfortable 5 = extremely comfortable)
| Subgroup analysis Baseline Comfort with POCUS applications | | | | |
| --- | --- | --- | --- | --- |
| Percent who felt at least "4-somewhat comfortable" with POCUS | Attendings (66) | Residents (23) | | p |
| procedural POCUS | 1.52% (1) | 0% (0) | | >0.99 |
| diagnostic POCUS | 0% (0) | 17% (4) | | 0.004 |
| | | | | |
| Percent who felt at least "4-somewhat comfortable" with POCUS | Inpatient (15) | Mixed (22) | Outpatient (29) | p |
| procedural POCUS | 6.67% (1) | 0% (0) | 0% (0) | 0.23 |
| diagnostic POCUS | 0% (0) | 0% (0) | 0% (0) | >0.99 |
| | | | | |

## Slide 2
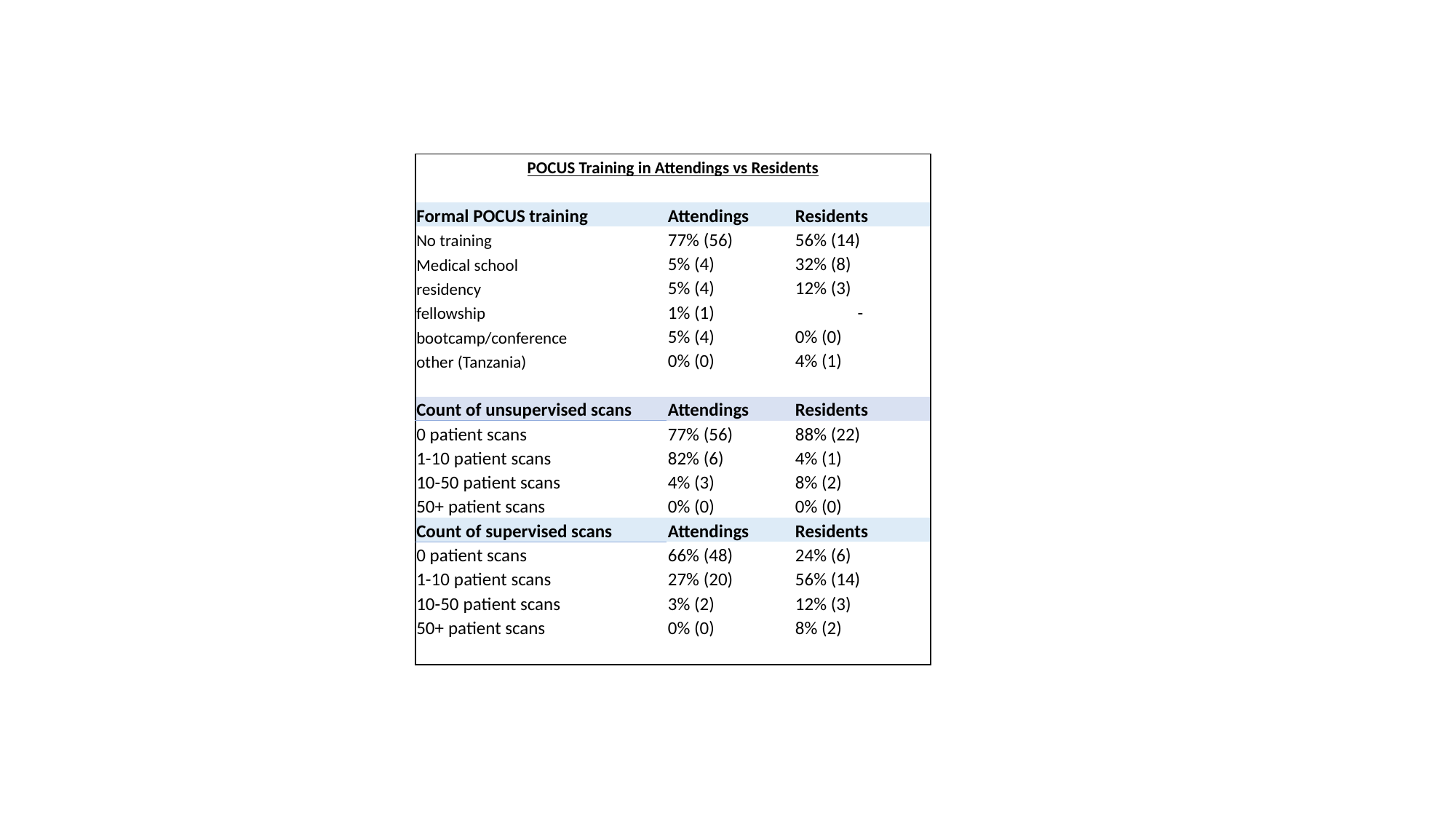

| POCUS Training in Attendings vs Residents | | |
| --- | --- | --- |
| | | |
| Formal POCUS training | Attendings | Residents |
| No training | 77% (56) | 56% (14) |
| Medical school | 5% (4) | 32% (8) |
| residency | 5% (4) | 12% (3) |
| fellowship | 1% (1) | - |
| bootcamp/conference | 5% (4) | 0% (0) |
| other (Tanzania) | 0% (0) | 4% (1) |
| | | |
| Count of unsupervised scans | Attendings | Residents |
| 0 patient scans | 77% (56) | 88% (22) |
| 1-10 patient scans | 82% (6) | 4% (1) |
| 10-50 patient scans | 4% (3) | 8% (2) |
| 50+ patient scans | 0% (0) | 0% (0) |
| Count of supervised scans | Attendings | Residents |
| 0 patient scans | 66% (48) | 24% (6) |
| 1-10 patient scans | 27% (20) | 56% (14) |
| 10-50 patient scans | 3% (2) | 12% (3) |
| 50+ patient scans | 0% (0) | 8% (2) |
| | | |

## Slide 3
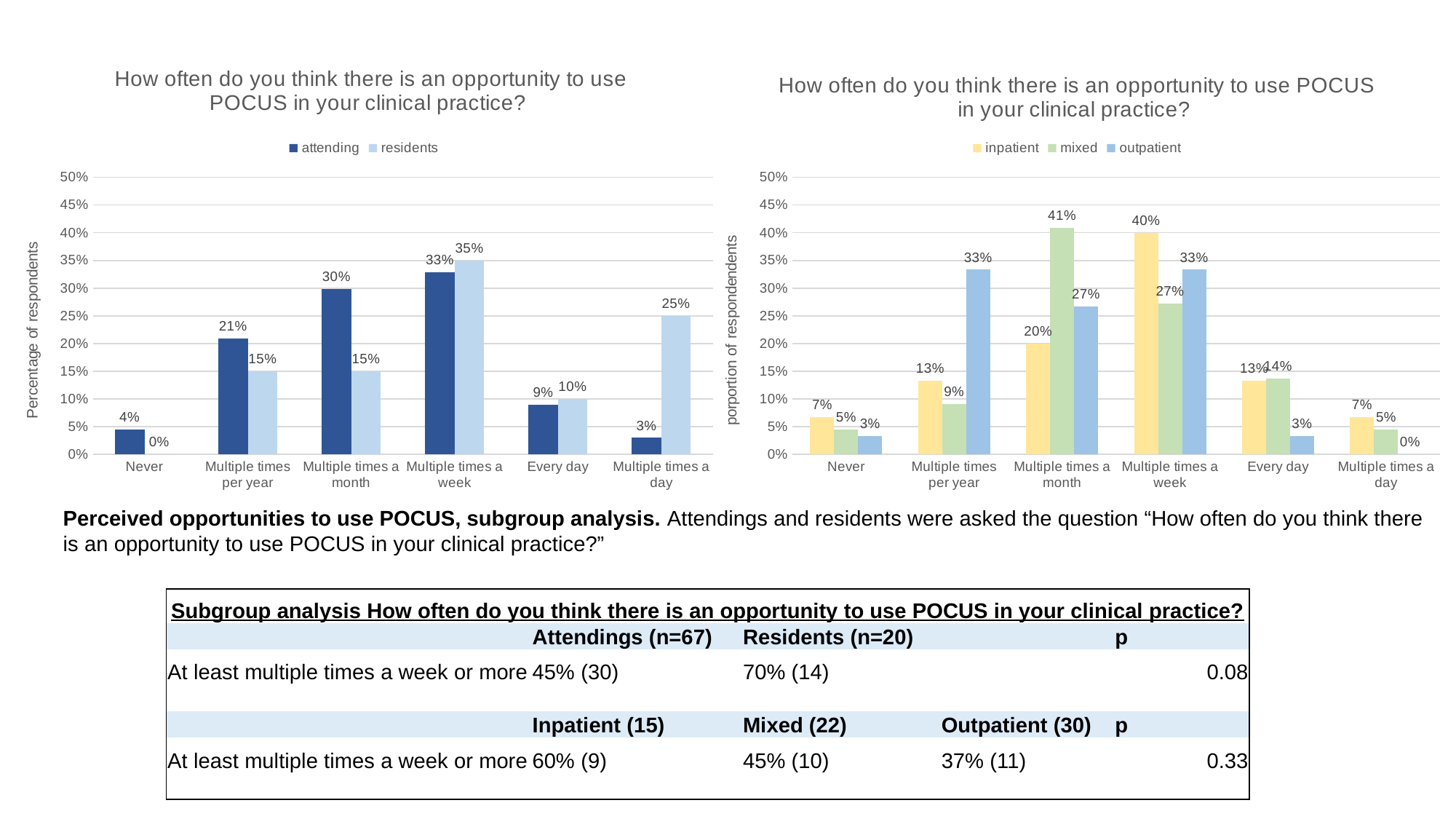

### Chart: How often do you think there is an opportunity to use POCUS in your clinical practice?
| Category | | |
|---|---|---|
| Never | 0.04477611940298507 | 0.0 |
| Multiple times per year | 0.208955223880597 | 0.15 |
| Multiple times a month | 0.29850746268656714 | 0.15 |
| Multiple times a week | 0.3283582089552239 | 0.35 |
| Every day | 0.08955223880597014 | 0.1 |
| Multiple times a day | 0.029850746268656716 | 0.25 |
### Chart: How often do you think there is an opportunity to use POCUS in your clinical practice?
| Category | | | |
|---|---|---|---|
| Never | 0.06666666666666667 | 0.045454545454545456 | 0.03333333333333333 |
| Multiple times per year | 0.13333333333333333 | 0.09090909090909091 | 0.3333333333333333 |
| Multiple times a month | 0.2 | 0.4090909090909091 | 0.26666666666666666 |
| Multiple times a week | 0.4 | 0.2727272727272727 | 0.3333333333333333 |
| Every day | 0.13333333333333333 | 0.13636363636363635 | 0.03333333333333333 |
| Multiple times a day | 0.06666666666666667 | 0.045454545454545456 | 0.0 |Perceived opportunities to use POCUS, subgroup analysis. Attendings and residents were asked the question “How often do you think there is an opportunity to use POCUS in your clinical practice?”
| Subgroup analysis How often do you think there is an opportunity to use POCUS in your clinical practice? | | | | |
| --- | --- | --- | --- | --- |
| | Attendings (n=67) | Residents (n=20) | | p |
| At least multiple times a week or more | 45% (30) | 70% (14) | | 0.08 |
| | | | | |
| | Inpatient (15) | Mixed (22) | Outpatient (30) | p |
| At least multiple times a week or more | 60% (9) | 45% (10) | 37% (11) | 0.33 |
| | | | | |

## Slide 4
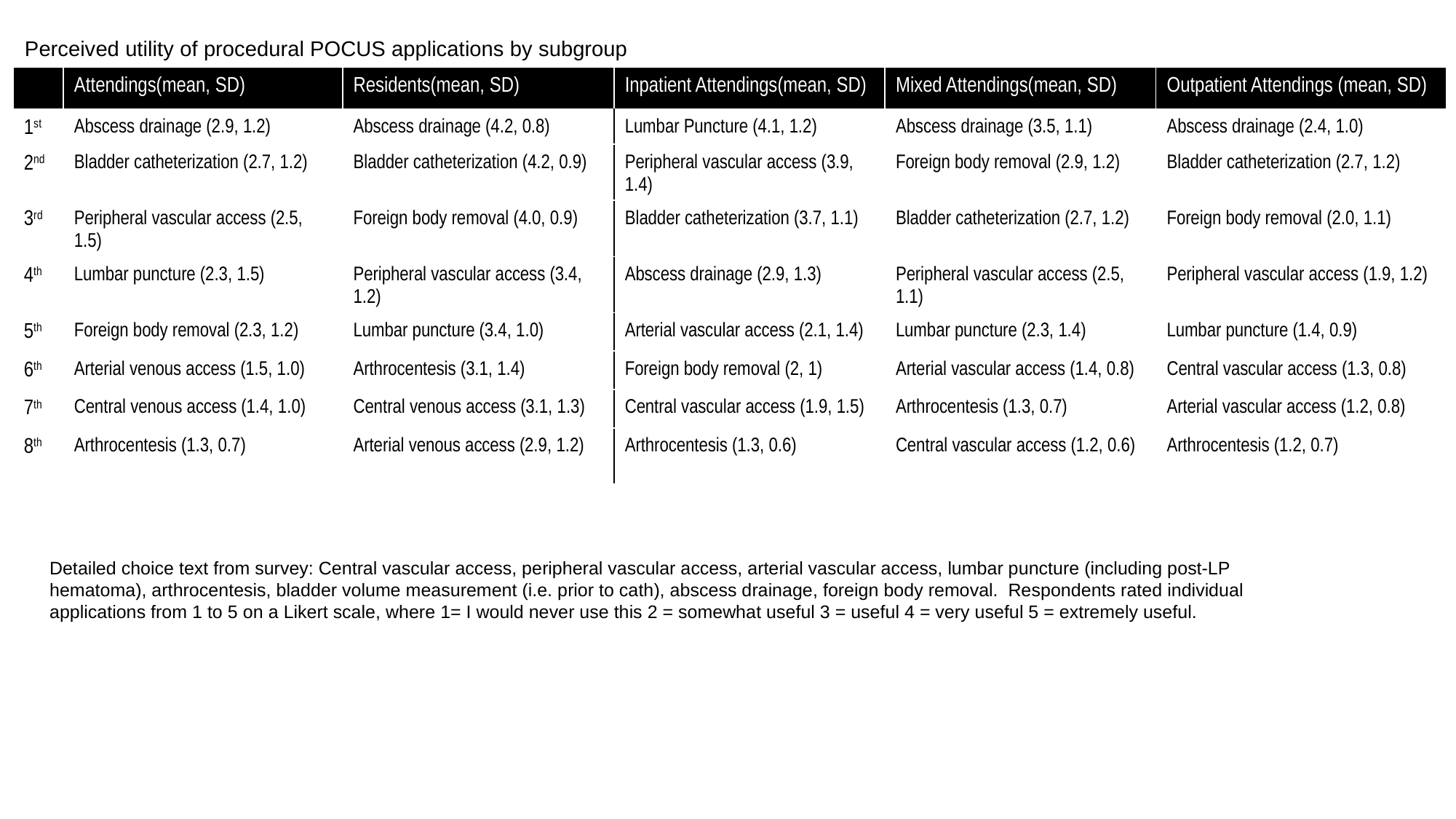

Perceived utility of procedural POCUS applications by subgroup
| | Attendings(mean, SD) | Residents(mean, SD) | Inpatient Attendings(mean, SD) | Mixed Attendings(mean, SD) | Outpatient Attendings (mean, SD) |
| --- | --- | --- | --- | --- | --- |
| 1st | Abscess drainage (2.9, 1.2) | Abscess drainage (4.2, 0.8) | Lumbar Puncture (4.1, 1.2) | Abscess drainage (3.5, 1.1) | Abscess drainage (2.4, 1.0) |
| 2nd | Bladder catheterization (2.7, 1.2) | Bladder catheterization (4.2, 0.9) | Peripheral vascular access (3.9, 1.4) | Foreign body removal (2.9, 1.2) | Bladder catheterization (2.7, 1.2) |
| 3rd | Peripheral vascular access (2.5, 1.5) | Foreign body removal (4.0, 0.9) | Bladder catheterization (3.7, 1.1) | Bladder catheterization (2.7, 1.2) | Foreign body removal (2.0, 1.1) |
| 4th | Lumbar puncture (2.3, 1.5) | Peripheral vascular access (3.4, 1.2) | Abscess drainage (2.9, 1.3) | Peripheral vascular access (2.5, 1.1) | Peripheral vascular access (1.9, 1.2) |
| 5th | Foreign body removal (2.3, 1.2) | Lumbar puncture (3.4, 1.0) | Arterial vascular access (2.1, 1.4) | Lumbar puncture (2.3, 1.4) | Lumbar puncture (1.4, 0.9) |
| 6th | Arterial venous access (1.5, 1.0) | Arthrocentesis (3.1, 1.4) | Foreign body removal (2, 1) | Arterial vascular access (1.4, 0.8) | Central vascular access (1.3, 0.8) |
| 7th | Central venous access (1.4, 1.0) | Central venous access (3.1, 1.3) | Central vascular access (1.9, 1.5) | Arthrocentesis (1.3, 0.7) | Arterial vascular access (1.2, 0.8) |
| 8th | Arthrocentesis (1.3, 0.7) | Arterial venous access (2.9, 1.2) | Arthrocentesis (1.3, 0.6) | Central vascular access (1.2, 0.6) | Arthrocentesis (1.2, 0.7) |
Detailed choice text from survey: Central vascular access, peripheral vascular access, arterial vascular access, lumbar puncture (including post-LP hematoma), arthrocentesis, bladder volume measurement (i.e. prior to cath), abscess drainage, foreign body removal. Respondents rated individual applications from 1 to 5 on a Likert scale, where 1= I would never use this 2 = somewhat useful 3 = useful 4 = very useful 5 = extremely useful.

## Slide 5
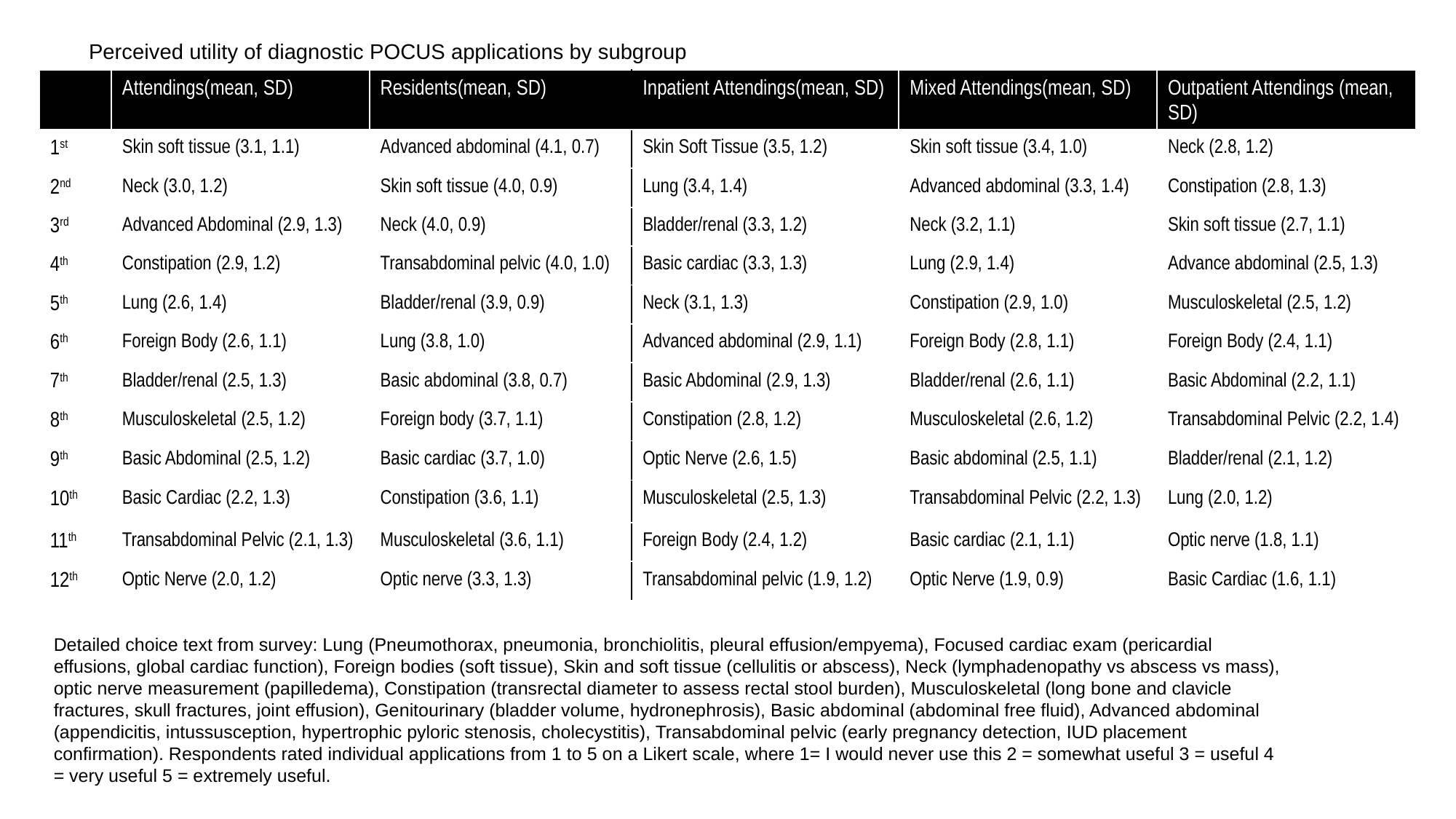

Perceived utility of diagnostic POCUS applications by subgroup
| | Attendings(mean, SD) | Residents(mean, SD) | Inpatient Attendings(mean, SD) | Mixed Attendings(mean, SD) | Outpatient Attendings (mean, SD) |
| --- | --- | --- | --- | --- | --- |
| 1st | Skin soft tissue (3.1, 1.1) | Advanced abdominal (4.1, 0.7) | Skin Soft Tissue (3.5, 1.2) | Skin soft tissue (3.4, 1.0) | Neck (2.8, 1.2) |
| 2nd | Neck (3.0, 1.2) | Skin soft tissue (4.0, 0.9) | Lung (3.4, 1.4) | Advanced abdominal (3.3, 1.4) | Constipation (2.8, 1.3) |
| 3rd | Advanced Abdominal (2.9, 1.3) | Neck (4.0, 0.9) | Bladder/renal (3.3, 1.2) | Neck (3.2, 1.1) | Skin soft tissue (2.7, 1.1) |
| 4th | Constipation (2.9, 1.2) | Transabdominal pelvic (4.0, 1.0) | Basic cardiac (3.3, 1.3) | Lung (2.9, 1.4) | Advance abdominal (2.5, 1.3) |
| 5th | Lung (2.6, 1.4) | Bladder/renal (3.9, 0.9) | Neck (3.1, 1.3) | Constipation (2.9, 1.0) | Musculoskeletal (2.5, 1.2) |
| 6th | Foreign Body (2.6, 1.1) | Lung (3.8, 1.0) | Advanced abdominal (2.9, 1.1) | Foreign Body (2.8, 1.1) | Foreign Body (2.4, 1.1) |
| 7th | Bladder/renal (2.5, 1.3) | Basic abdominal (3.8, 0.7) | Basic Abdominal (2.9, 1.3) | Bladder/renal (2.6, 1.1) | Basic Abdominal (2.2, 1.1) |
| 8th | Musculoskeletal (2.5, 1.2) | Foreign body (3.7, 1.1) | Constipation (2.8, 1.2) | Musculoskeletal (2.6, 1.2) | Transabdominal Pelvic (2.2, 1.4) |
| 9th | Basic Abdominal (2.5, 1.2) | Basic cardiac (3.7, 1.0) | Optic Nerve (2.6, 1.5) | Basic abdominal (2.5, 1.1) | Bladder/renal (2.1, 1.2) |
| 10th | Basic Cardiac (2.2, 1.3) | Constipation (3.6, 1.1) | Musculoskeletal (2.5, 1.3) | Transabdominal Pelvic (2.2, 1.3) | Lung (2.0, 1.2) |
| 11th | Transabdominal Pelvic (2.1, 1.3) | Musculoskeletal (3.6, 1.1) | Foreign Body (2.4, 1.2) | Basic cardiac (2.1, 1.1) | Optic nerve (1.8, 1.1) |
| 12th | Optic Nerve (2.0, 1.2) | Optic nerve (3.3, 1.3) | Transabdominal pelvic (1.9, 1.2) | Optic Nerve (1.9, 0.9) | Basic Cardiac (1.6, 1.1) |
Detailed choice text from survey: Lung (Pneumothorax, pneumonia, bronchiolitis, pleural effusion/empyema), Focused cardiac exam (pericardial effusions, global cardiac function), Foreign bodies (soft tissue), Skin and soft tissue (cellulitis or abscess), Neck (lymphadenopathy vs abscess vs mass), optic nerve measurement (papilledema), Constipation (transrectal diameter to assess rectal stool burden), Musculoskeletal (long bone and clavicle fractures, skull fractures, joint effusion), Genitourinary (bladder volume, hydronephrosis), Basic abdominal (abdominal free fluid), Advanced abdominal (appendicitis, intussusception, hypertrophic pyloric stenosis, cholecystitis), Transabdominal pelvic (early pregnancy detection, IUD placement confirmation). Respondents rated individual applications from 1 to 5 on a Likert scale, where 1= I would never use this 2 = somewhat useful 3 = useful 4 = very useful 5 = extremely useful.

## Slide 6
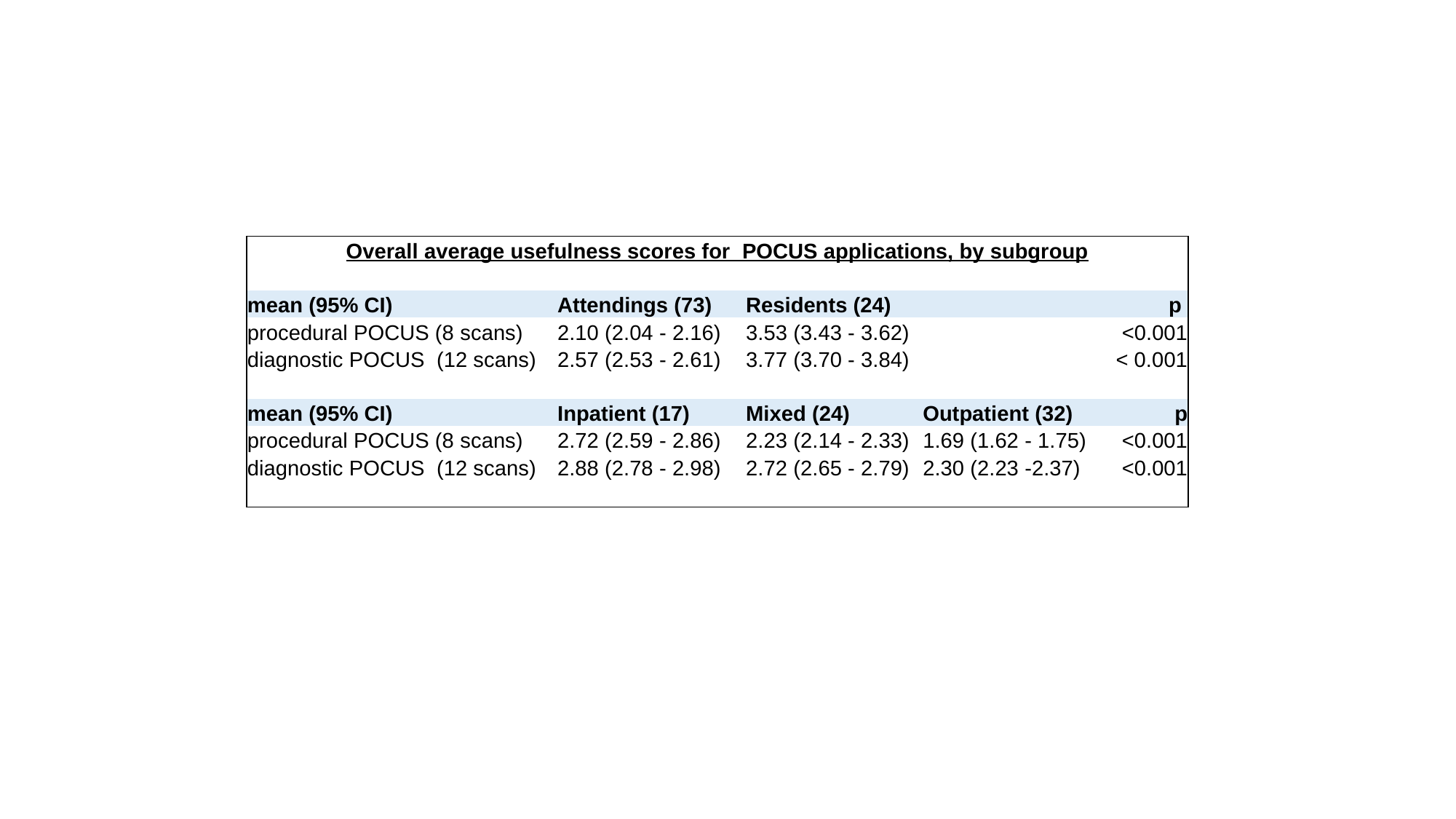

| Overall average usefulness scores for POCUS applications, by subgroup | | | | |
| --- | --- | --- | --- | --- |
| | | | | |
| mean (95% CI) | Attendings (73) | Residents (24) | | p |
| procedural POCUS (8 scans) | 2.10 (2.04 - 2.16) | 3.53 (3.43 - 3.62) | | <0.001 |
| diagnostic POCUS (12 scans) | 2.57 (2.53 - 2.61) | 3.77 (3.70 - 3.84) | | < 0.001 |
| | | | | |
| mean (95% CI) | Inpatient (17) | Mixed (24) | Outpatient (32) | p |
| procedural POCUS (8 scans) | 2.72 (2.59 - 2.86) | 2.23 (2.14 - 2.33) | 1.69 (1.62 - 1.75) | <0.001 |
| diagnostic POCUS (12 scans) | 2.88 (2.78 - 2.98) | 2.72 (2.65 - 2.79) | 2.30 (2.23 -2.37) | <0.001 |
| | | | | |

## Slide 7
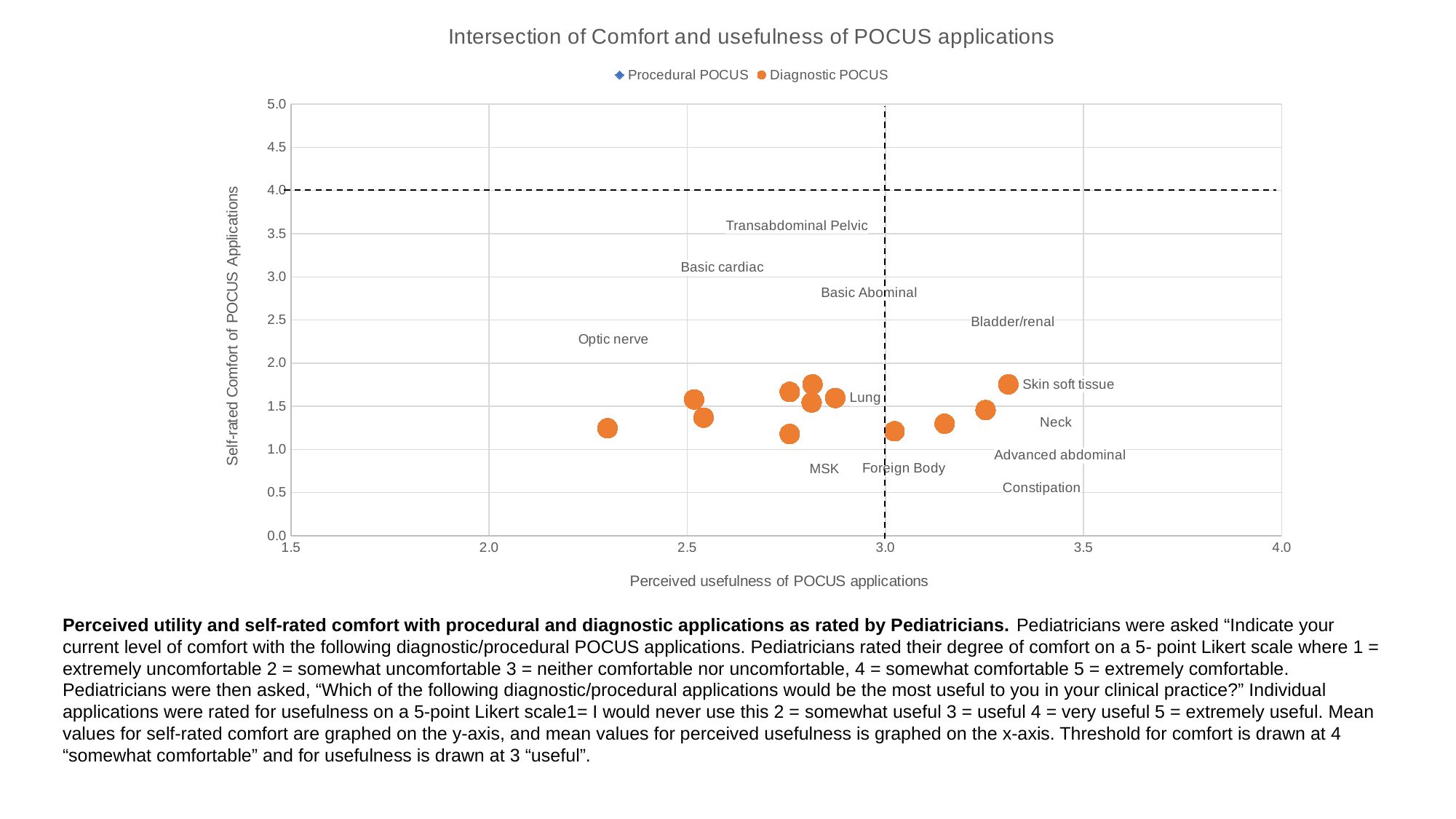

### Chart: Intersection of Comfort and usefulness of POCUS applications
| Category | | |
|---|---|---|Perceived utility and self-rated comfort with procedural and diagnostic applications as rated by Pediatricians. Pediatricians were asked “Indicate your current level of comfort with the following diagnostic/procedural POCUS applications. Pediatricians rated their degree of comfort on a 5- point Likert scale where 1 = extremely uncomfortable 2 = somewhat uncomfortable 3 = neither comfortable nor uncomfortable, 4 = somewhat comfortable 5 = extremely comfortable. Pediatricians were then asked, “Which of the following diagnostic/procedural applications would be the most useful to you in your clinical practice?” Individual applications were rated for usefulness on a 5-point Likert scale1= I would never use this 2 = somewhat useful 3 = useful 4 = very useful 5 = extremely useful. Mean values for self-rated comfort are graphed on the y-axis, and mean values for perceived usefulness is graphed on the x-axis. Threshold for comfort is drawn at 4 “somewhat comfortable” and for usefulness is drawn at 3 “useful”.
